# Predictors of postpandemic preparedness for special pathogens

**DOI:** 10.1017/ash.2024.393

**Published:** 2024-09-09

**Authors:** Morgan M. Kuhnly, Caitlin M. Adams Barker, Kathleen O. Stewart, Justin J. Kim

**Affiliations:** 1 Collaborative Healthcare-associated Infection Prevention Program, Dartmouth Hitchcock Medical Center, Lebanon, NH, USA; 2 Quality Assurance and Safety, Dartmouth Hitchcock Medical Center, Lebanon, NH, USA; 3 Section of Infectious Disease and International Health, Dartmouth Hitchcock Medical Center, Lebanon, NH, USA; 4 Geisel School of Medicine at Dartmouth College, Hanover, NH, USA

## Abstract

In this survey of 31 hospitals, large metropolitan facilities had a 9.5-fold odds of reporting preparedness for special pathogens; hospitals with special pathogens teams had a 14.3-fold odds of reporting preparedness for special pathogens. In the postpandemic world, healthcare institutions must invest in special pathogen responses to maximize patient safety.

## Introduction

After the coronavirus disease 2019 (COVID-19) pandemic, the state of postpandemic preparedness for other emerging infectious diseases is uncertain for many institutions.^1^ However, other non-COVID special pathogens such as Ebola, Marburg, Lassa fever, Middle East respiratory syndrome/severe acute respiratory syndrome, Nipah, Zika, and Rift Valley fever have not decreased.^2^ As healthcare facilities may not have had time or resources to revisit old response plans, many facilities and their patients may be at risk.^3^ In the postpandemic world, healthcare institutions must continue to invest in their special pathogen responses to maximize care for patients at all points of entry.

Preparedness is multifaceted and can be assessed at the level of individuals, departments, hospitals, communities, and even countries.^4^ Few, if any, studies of institutional preparedness for special pathogens are available in the infection control literature, and there are even fewer from the post-COVID-19 era.^5^ The purpose of this study was to ascertain the predictors of perceived preparedness for postpandemic special pathogens at the facility level.

## Methods

### Study design and participants

We conducted an exploratory study through the Society for Healthcare Epidemiology of America (SHEA) Research Network, a consortium of over 100 unique healthcare facilities collaborating on multicenter research projects in healthcare epidemiology and antimicrobial stewardship. The survey questionnaire was investigator-developed and reviewed for face validity by our infection prevention team and hospital epidemiologist. The survey questionnaire was distributed to the US members of the SHEA Research Network in October 2023 (Supplemental Figure 1). This study was determined to be nonhuman subject research by the Dartmouth Hitchcock Medical Center Institutional Review Board.

### Exposure, outcomes, and covariates

The primary outcome was the facility’s preparedness for special pathogens. We defined preparedness using answers to the question: “How would you assess your facility’s global preparedness toward infectious threats (ability to minimize and detect potential infectious threats, minimizing spread)?” We classified responses of “strong” or “adequate” as prepared and “neutral” or “limited” as unprepared.

Additional covariates included demographics including bed size (ie, <200, 201–400, 401–600, 601–800, 801–1,000, and >1,000 beds), type of facility (academic vs nonacademic medical center), and National Center for Health Statistics Urban-Rural Classification Scheme for Counties.^6^ We dichotomized the classification to large metro (ie, large fringe or large central metro) and other (ie, medium metro, small metro, micropolitan, or noncore).

Additionally, we evaluated prepandemic (ie, before March 11, 2020) predictors, postpandemic (ie, after May 11, 2023) predictors, overall support, and the type of facility (ie, frontline, assessment, or treatment center) (Table 1).

**Table 1.** Special pathogen postpandemic preparedness

Characteristic	Total n = 31	Prepared n = 23	Not prepared n = 8	*P*-value^a^
***Demographics***				
Bed size				0.34
<201	3 (10%)	2 (9%)	1 (13%)	
201–400	6 (19%)	3 (13%)	3 (38%)	
401–600	11 (35%)	9 (39%)	2 (25%)	
601–800	4 (13%)	2 (9%)	2 (25%)	
801–1,000	5 (16%)	5 (22%)	0 (0%)	
>1,000	2 (6%)	2 (9%)	0 (0%)	
Academic medical center	19 (61%)	16 (70%)	3 (38%)	0.21
IP staffing per 100 inpatient beds, mean (SD)	1.32 (0.62)	1.25 (0.57)	1.48 (0.75)	0.38
***National for Center Health Statistics Urban-Rural Classification Scheme for Counties***				
Large central and large fringe metro	20 (65%)	18 (78%)	2 (25%)	0.012
Large central	13 (42%)	12 (52%)	1 (13%)	0.095
***Prepandemic***				
Facility determination				0.074
Frontline facility	7 (23%)	3 (13%)	4 (50%)	
Assessment facility	13 (42%)	10 (43%)	3 (38%)	
Treatment facility	11 (35%)	10 (43%)	1 (13%)	
Prepandemic screening^b^	7 (23%)	6 (26%)	1 (13%)	0.64
Cared for suspected or confirmed VHF patients in the past 5 years	27 (87%)	4 (17%)	0 (0%)	0.55
Staffed a biocontainment unit	14 (45%)	11 (48%)	3 (38%)	0.70
Staffed a special pathogens team	24 (77%)	20 (87%)	4 (50%)	0.053
***Postpandemic***				
Facility determination				0.21
Frontline facility	8 (26%)	4 (17%)	4 (50%)	
Assessment facility	13 (42%)	10 (43%)	3 (38%)	
Treatment facility	10 (32%)	9 (39%)	1 (13%)	
Postpandemic screening^b^	9 (29%)	9 (39%)	0 (0%)	0.068
Staffed a biocontainment unit	11 (35%)	9 (39%)	2 (25%)	0.68
Staffed a special pathogens team	17 (55%)	16 (70%)	1 (13%)	0.011
Engagement with regional treatment center^c^	10 (32%)	9 (39%)	1 (13%)	0.22
Engagement from state^c^	8 (26%)	8 (35%)	0 (0%)	0.076
Engagement from system^c^	8 (26%)	8 (35%)	0 (0%)	0.076
Engagement from organizational leadership^c^	9 (29%)	9 (39%)	0 (0%)	0.068
Financial support for PPE	17 (55%)	13 (57%)	4 (50%)	1.00
Financial support for staffing	12 (39%)	11 (48%)	1 (13%)	0.11
Appropriately stocked PPE^b^	26 (84%)	21 (91%)	5 (63%)	0.093
Lab capacity for malaria testing	29 (94%)	22 (96%)	7 (88%)	0.46
Transport plan	28 (90%)	23 (100%)	5 (63%)	0.012
***Overall preparedness***				
Facility felt more prepared				0.69
Prepandemic	12 (39%)	8 (35%)	4 (50%)	
Postpandemic	8 (26%)	7 (30%)	1 (13%)	
Equal	11 (35%)	8 (35%)	3 (28%)	

Note. VHF, viral hemorrhagic fever; PPE, personal protective equipment; SD, standard deviation.

a Fisher exact tests were used to compare proportions among categorical variables; *t* test was used to compare continuous variables.

b Response of “Almost always.”

c Response of “Very engaged.”

### Statistical analysis

We compared the distribution of covariates between prepared and unprepared facilities comparing proportions for categorical variables using the Fisher exact tests as appropriate. We used logistic regression to assess the relative contribution of significant associations in the bivariate analysis (ie, *P* < .05). We used correlation coefficients to ensure that highly correlated covariates would not be included in the final model. We used STATA 15.1 (StataCorp, College Station, Texas, USA) for all statistical analyses.

## Results

We received complete responses from 31 to 94 (33%) eligible facilities in the United States. Demographics including bed size and type of facility (ie, academic medical center vs nonacademic medical center) were not significantly different between prepared and unprepared facilities. Compared to survey respondents, nonrespondents had similar bed sizes and types of facilities (Supplemental Table 2).

Twenty-three of 31 (74%) respondents reported that their hospitals were prepared for special pathogens. A greater proportion of prepared hospitals were from large metropolitan areas (18 of 23 [78%] vs 2 of 8 [25%], *P* = .012), reported staffing a special pathogens team (16 of 23 [70%] vs 1 of 8 [13%], *P* = .01), and having a transport team (23 of 23 [100%] vs 5 of 8 [63%], *P* = .01) compared to unprepared hospitals. Having a special pathogens team was highly correlated with having a transport team (*P* = .99).

By logistic regression, hospitals from large metropolitan facilities had 10.8-fold odds (95% CI, 1.64–70.9) of reporting preparedness for special pathogens compared to other facilities. Hospitals with a special pathogens team had a 16.0-fold odds (95% CI, 1.64–156) of reporting preparedness for special pathogens compared to hospitals without a special pathogens team. By multivariate logistic regression where preparedness was a function of being from a large metropolitan area and having a special pathogens team, these odds were both still significant at 9.5 (95% CI, 1.12–81.3) and 14.3 (95% CI, 17.9–167), respectively.

## Discussion

To our knowledge, this is the first study in the infection control literature whose purpose is to ascertain predictors related to postpandemic preparedness for special pathogens. Having a special pathogens team and being located in a large metropolitan area were the strongest predictors of perceived preparedness. Having a special pathogens team could be a surrogate measure of institutional preparedness, while being located in a large metropolitan area could represent other factors key to program functionality and maintenance such as having the population to offset staffing shortages and turnover and the opportunity for informal collaboration with other institutions. Our work highlights the need for institutional buy-in as well as collaboration and resources outside the institution to help facilitate special pathogen preparedness, particularly in rural settings. Considering how many patients receive care in rural areas, the infection prevention and emergency preparedness community must strengthen preparedness in these healthcare settings.

The primary strength of this study was to provide insight into potential predictors of postpandemic preparedness from a variety of respondents and institutions. Our study had several limitations. This study did not consist of a simple random sample. The sample size was relatively small, and we were unable to adjust for additional confounders in our multivariate model. The response rate was low, which could have resulted in a selection bias, though there was no significant difference between the demographics of survey responders and nonresponders. Finally, we assumed that the responses of survey participants accurately reflected the perceptions of their institutions.

As institutions continue to recover from the COVID-19 pandemic, we see this study as the beginning of a series of important conversations whose goal is to understand effective strategies to address barriers needed to maintain special pathogens preparedness. Continued partnerships with all aspects of the special pathogen response continuum will be integral in maintaining hospital readiness and protecting patients and staff members alike.

## Supporting information

Kuhnly et al. supplementary material Kuhnly et al. supplementary material

## Supplementary material

For supplementary material accompanying this paper visit http://doi.org/10.1017/ash.2024.393. click here to view supplementary material

## References

[1] Henderson DK , Haessler S , Weber, DJ. The coronavirus disease 2019 (COVID-19) pandemic- looking back and looking forward. Infection Control & Hospital Epidemiology 2021;42:1245–1250. 34334144 10.1017/ice.2021.338

[2] Polgreen PH , Santibanez S , Koonin LM , et al. Infectious disease physician assessment of hospital preparedness for Ebola virus disease. Open Forum Infectious Diseases 2015;2:ofb087. 10.1093/ofid/ofv087 PMC4499670 26180836

[3] French G , Hulse M , Nguyen, D , et al. Impact of hospital strain on excess deaths during the COVID-19 pandemic-united states. Morbidity and Mortality Weekly Report 2021;70:1613–16161. 34793414 10.15585/mmwr.mm7046a5 PMC8601411

[4] Lee JM , Jansen R , Sanderson K , et al. Public health emergency preparedness for infectious disease emergencies: a scoping review of recent evidence. BMC Public Health 2023;23:420. 36864415 10.1186/s12889-023-15313-7 PMC9979131

[5] Rebmann T , Wilson R , Lapointe S , et al. Hospital infectious disease emergency preparedness: a 2007 survey of infection control professionals. American Journal of Infection Control 2008;37:1–8. 19081162 10.1016/j.ajic.2008.02.007

[6] Data access – urban-rural classification scheme for counties. Published 2017. Centers for Disease Control and Prevention. https://www.cdc.gov/nchs/data_access/urban_rural.htm. Accessed February 5, 2024.

