# Supplementary material for: Predictors of postpandemic preparedness for special pathogens
Source: Antimicrob Steward Healthc Epidemiol. 2024 Sep 9;4(1):e122. doi: 10.1017/ash.2024.393 (PMC11384164; doi:10.1017/ash.2024.393)
Supplement: Kuhnly et al. supplementary material [file S2732494X24003930sup001.docx]

**Supplemental Figure 1: Questionnaire**


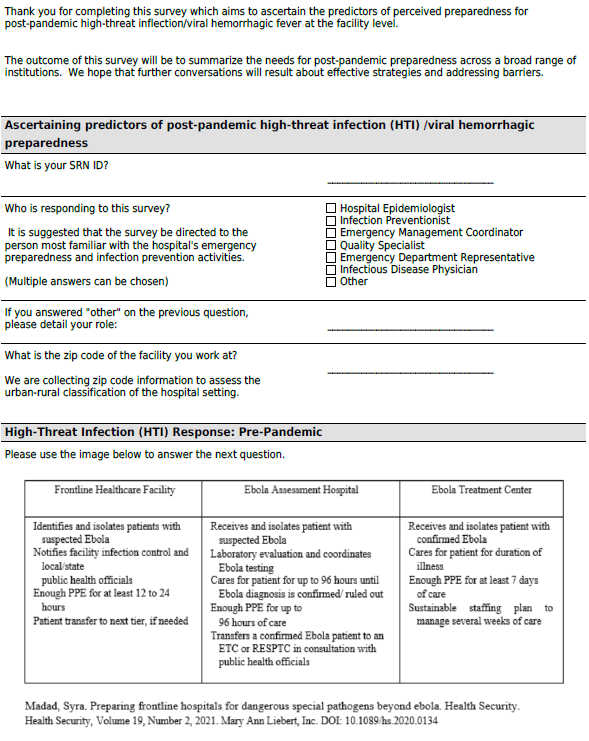


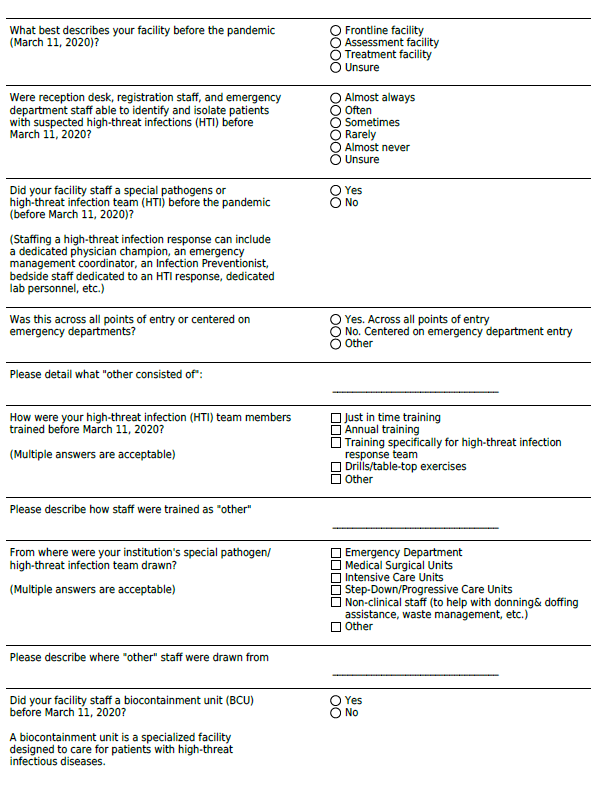


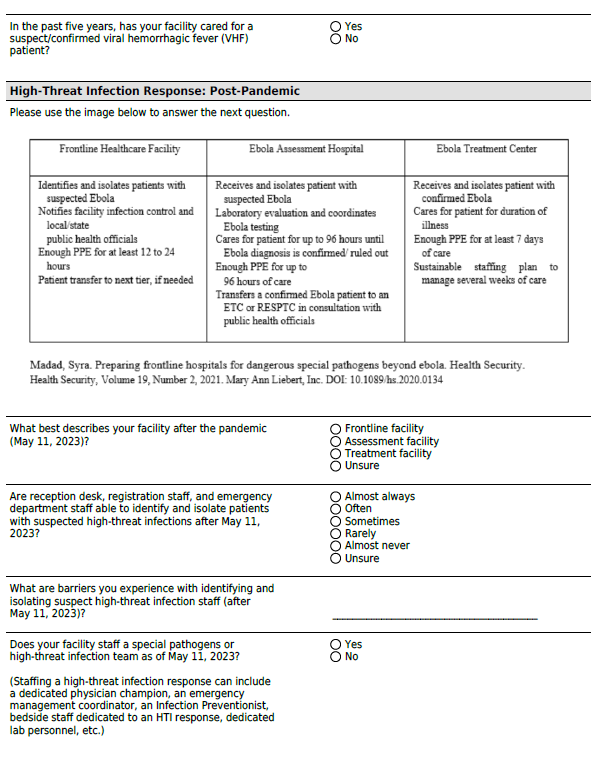


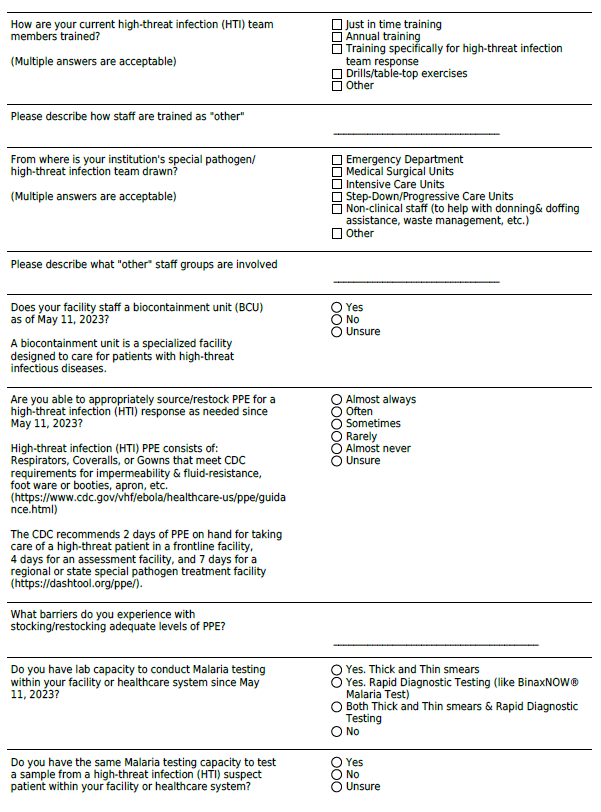


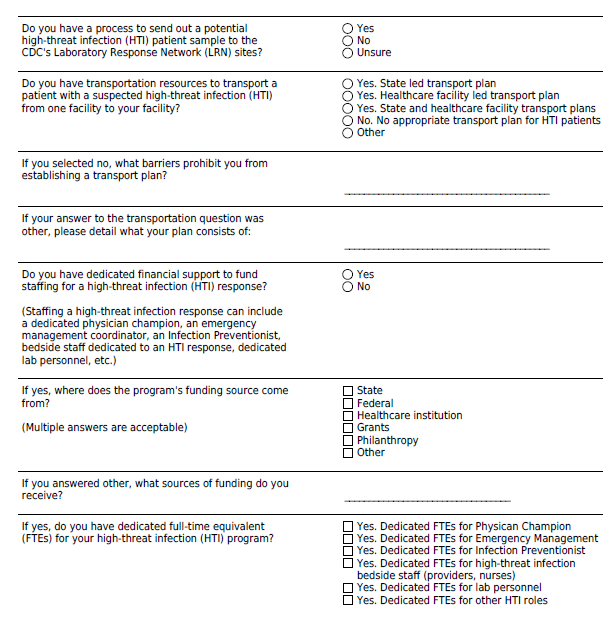


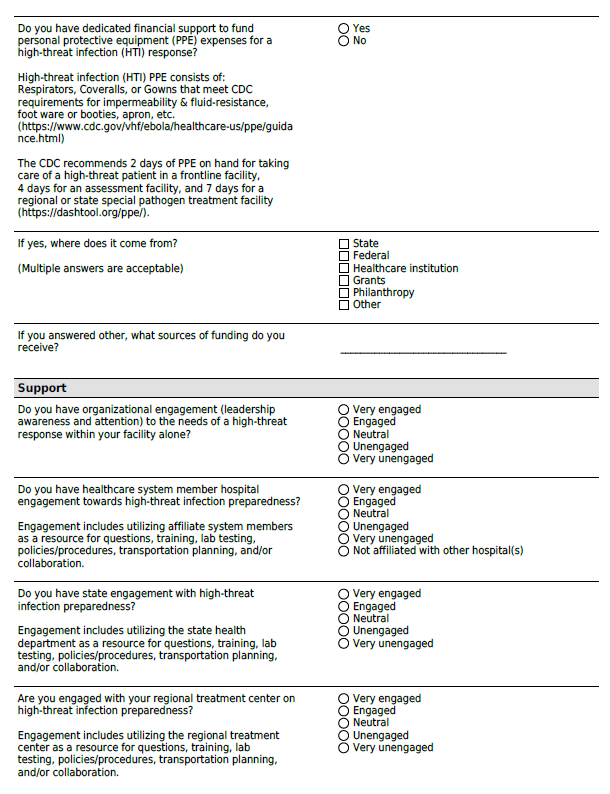


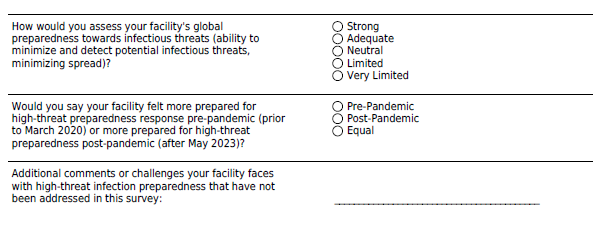


| **Supplemental Table 1: Demographics of Survey Respondents versus Non-Respondents** | | | |
| --- | --- | --- | --- |
|  | **Respondent**  **n=31** | **Non-Respondent**  **n=63** | **p-value^1^** |
| Bed Size |  |  |  |
| <201 | 3 (10%) | 7 (11%) | 0.76 |
| 201-400 | 6 (19%) | 8 (13%) |  |
| 401-600 | 11 (35%) | 15 (24%) |  |
| 601-800 | 4 (13%) | 13 (21%) |  |
| 801-1000 | 5 (16%) | 13 (21%) |  |
| >1000 | 2 (6%) | 6 (10%) |  |
| Academic medical center | 19 (61%) | 49 (78%) | 0.093 |

^1^Fisher exact tests were used to compare proportions among categorical variables
